# An Introduction to Entomology; or Elements of the Natural History of Insects

**Published:** 1846-06

**Authors:** 


Art. XIV.—*An Introduction to Entomology; or Elements of the Natural History of Insects: comprising an account of noxious and useful Insects, of their metamorphoses, food, stratagems, habitations, societies, motions, noises, hybemations, instinct, &c., &c.* With plates. By William Kirby, M.A., F.R.S.&L.S., Rector of Barham, and William Spence, Esq., F.R.S.&L.S. From the 6th London edition, which was corrected and considerably enlarged. Philadelphia: Lea & Blanchard. 1846. 8v. pp. 600.

In the very entertaining book of travels, alluded to in our last number, styled the "Voyage of a Naturalist," the following amusing incidents are related by Mr. Darwin as having happened while he was in Chili: "One day, a German collector in natural history, of the name of Renous, called, and nearly at the same time an old Spanish lawyer. Renous, alluding to me, asked him what he thought of the King of England sending out a collector to their country to pick up lizards and beetles, and to break stones. The old gentleman thought seriously for some time, and then said: 'It is not well—*hay un gato encerrado aqui* (there is a cat shut up here). No man is so rich as to send out people to pick up such rubbish. I do not like it: if one of us were to go and do such things in England, do not you think the King of England would very soon send us out of his country?' Renous himself, two or three years before, left in a house at S. Fernando some caterpillars, under charge of a girl to feed, that they might turn into butterflies. This was rumored through the town, and at last the Padres and the Governor consulted together, and agreed it must be some heresy. Accordingly, when Renous returned, he was arrested."

Perhaps, some of our readers agree with the old lawyer, in his estimate of the value of entomology: if so, we are pretty sure of not receiving their thanks for introducing the above work to their acquaintance. We are well aware that the physician engaged in an engrossing practice, whether in town or country, has not much leisure for the perusal of books unconnected with his profession; but we know just as well, that while the few are thus immersed in business, the many have the command of more time than they are disposed to give to professional reading. How many are the hours wasted by nearly every young physician, waiting for practice—anxious, dreary hours, because unoccupied! Why not spend these hours in the study of such works as that of Kirby and Spence, wherein the physiologist, farmer, horticulturist, philosopher, and moralist, may find matter to instruct him. We have the authority of at least one wise man, that we may gain wisdom by studying the habits of the animals below us. "Go to the ant," are the well known words of Solomon, "thou sluggard, consider her ways and be wise; which, having neither captain, overseer, nor ruler, prepares her bread in the summer and gathers her food in the harvest."

It is wise in us to enlarge the sphere of our intellectual enjoyments; and by few things can we do so more effectually than by cultivating a taste for natural history. To the Padres and Governor of Chili it appeared a very senseless if not heretical business, to be hatching butterflies, and breaking stones; but for the man whose taste has been improved by study, there is a charm in every walk of nature—in the humblest insect tribes, volumes of instruction—'sermons in stones, and good in everything.' Does not medicine owe to the insect race a valuable remedy; and is it not to the same race that we are indebted for honey and for silk? But not to pursue these commonplaces, we close our notice of this volume on Entomology, which will be found by those who read it rich in curious information, by a short extract concerning the medical relations of insects, of which the authors say: "Had I addressed you a century ago, I could have made this an ample history. Amongst scores of infallible panaceas, I should have recommended the *woodlouse* as a solvent and aperient; powder of *silk-worm* for vertigo and convulsions; *millepeds* against the jaundice; *earwigs* to strengthen the nerves; powdered *scorpion* for the stone and gravel; *fly-water* for disorders in the eyes, and the *tick* for erysipelas. I should have prescribed *five gnats* as an excellent purge; *wasps as diuretics;* lady-birds for the colic and measles; the cockchafer for the bite of a mad dog and the plague; and ants and their acid I should have loudly praised as incomparable against leprosy and deafness, as strengthening the memory, and giving vigor and animation to the whole bodily frame. But these good times have long gone by. You would, I fear, laugh at my prescriptions notwithstanding the great authorities I could cite in their favor; and even doubt the efficacy of a more modern specific for toothache, promulgated by a learned Italian professor, who assures us that a finger once imbued with the juice of *Rhinobatus antiodontalgicus* will retain its power of curing this disease for a twelvemonth!"

This beats homœopathy.

Kirby & Spence, Carpenter, Miller, &c., may be found at the bookstores of Jas. Maxwell and F. W. Prescott, in this city.

